# Supplementary material for: Crosstalk in Facial EMG and Its Reduction Using ICA
Source: Sensors (Basel). 2023 Mar 2;23(5):2720. doi: 10.3390/s23052720 (PMC10007323; doi:10.3390/s23052720)
Supplement: Supplementary file 1 [file sensors-23-02720-s001.zip › Figure S1,2,3 Table S1,2.pdf]

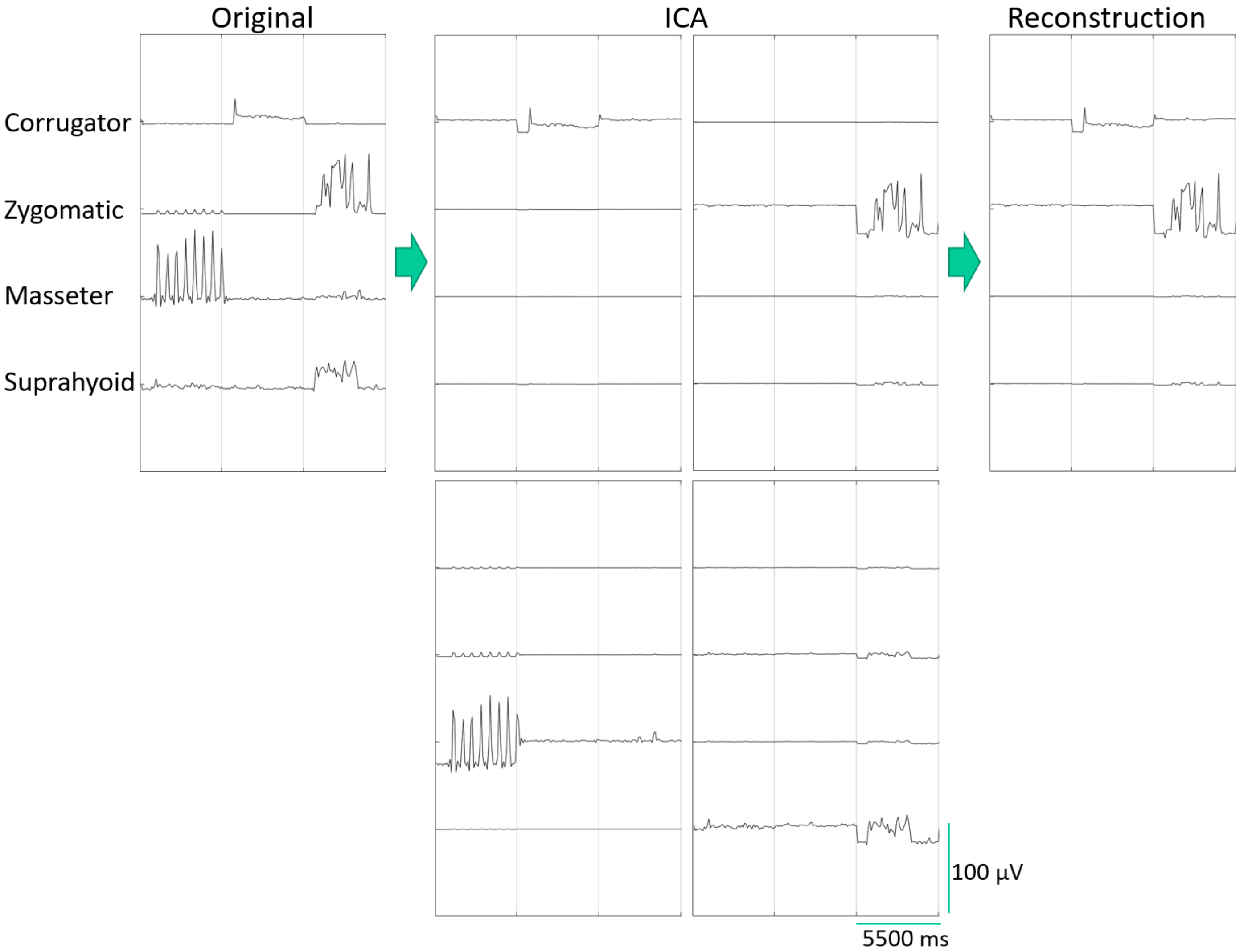

Figure S1. Independent component analysis (ICA) process. Data for three trials (total of 16.5 s; each trial contained 0.5-s pre- and 5-s post-stimulus periods) of a representative participant are shown. Original electromyography signals of the corrugator supercilii, zygomatic major, masseter, and suprahyoid muscles were subjected to ICA. To remove crosstalk arising from masseter and suprahyoid muscle activities, the signals were reconstructed using the independent components that exhibited the highest variance with respect to the corrugator supercilii and zygomatic major muscle activities.

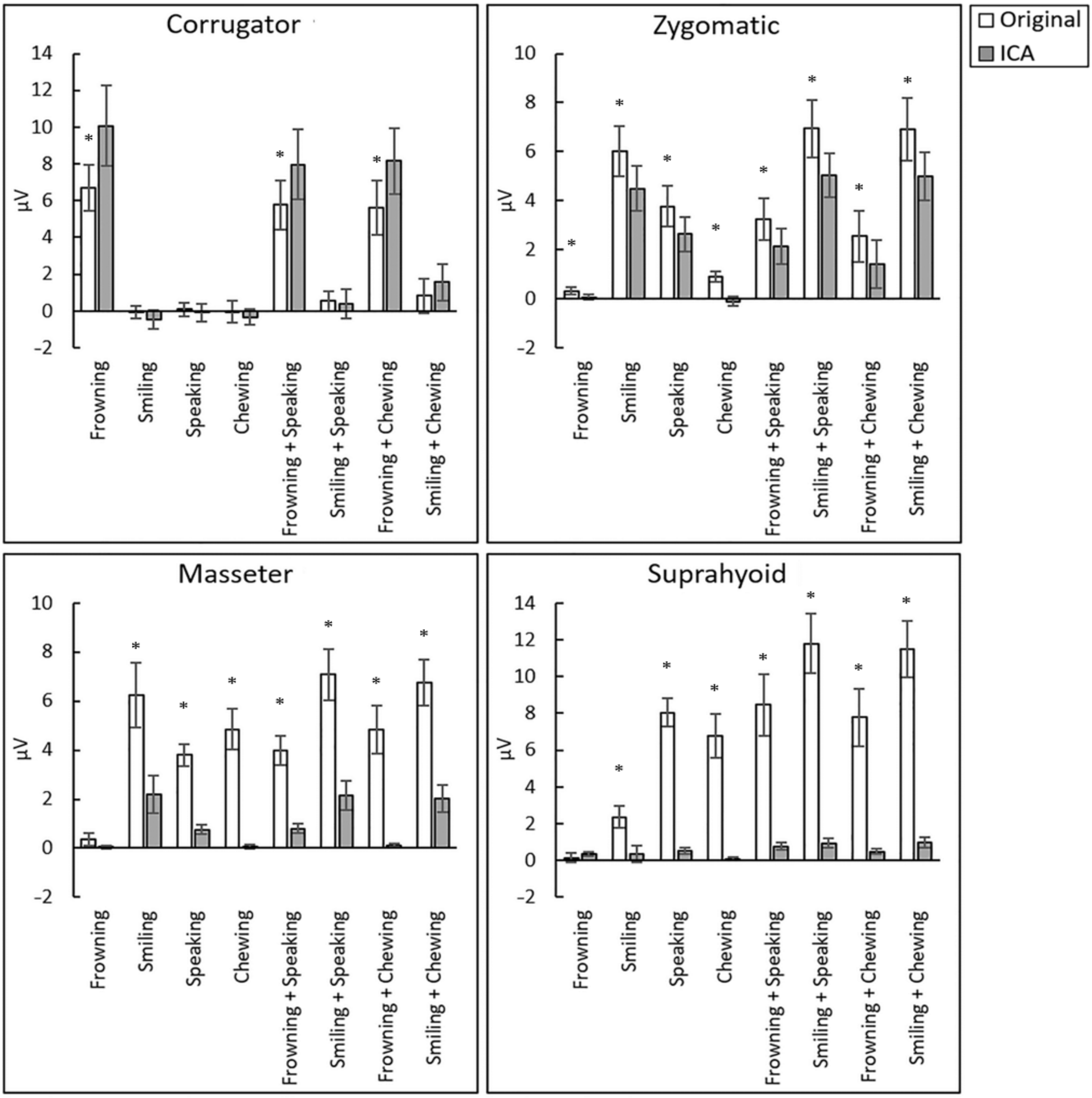

Figure S2. Mean ± standard error of original and independent component analysis (ICA)-reconstructed electromyography data indicating significant results of one-sample *t*-tests (vs. zero; two-tailed) of original electromyography signals. *, $p > 0.05$, Holm-corrected.

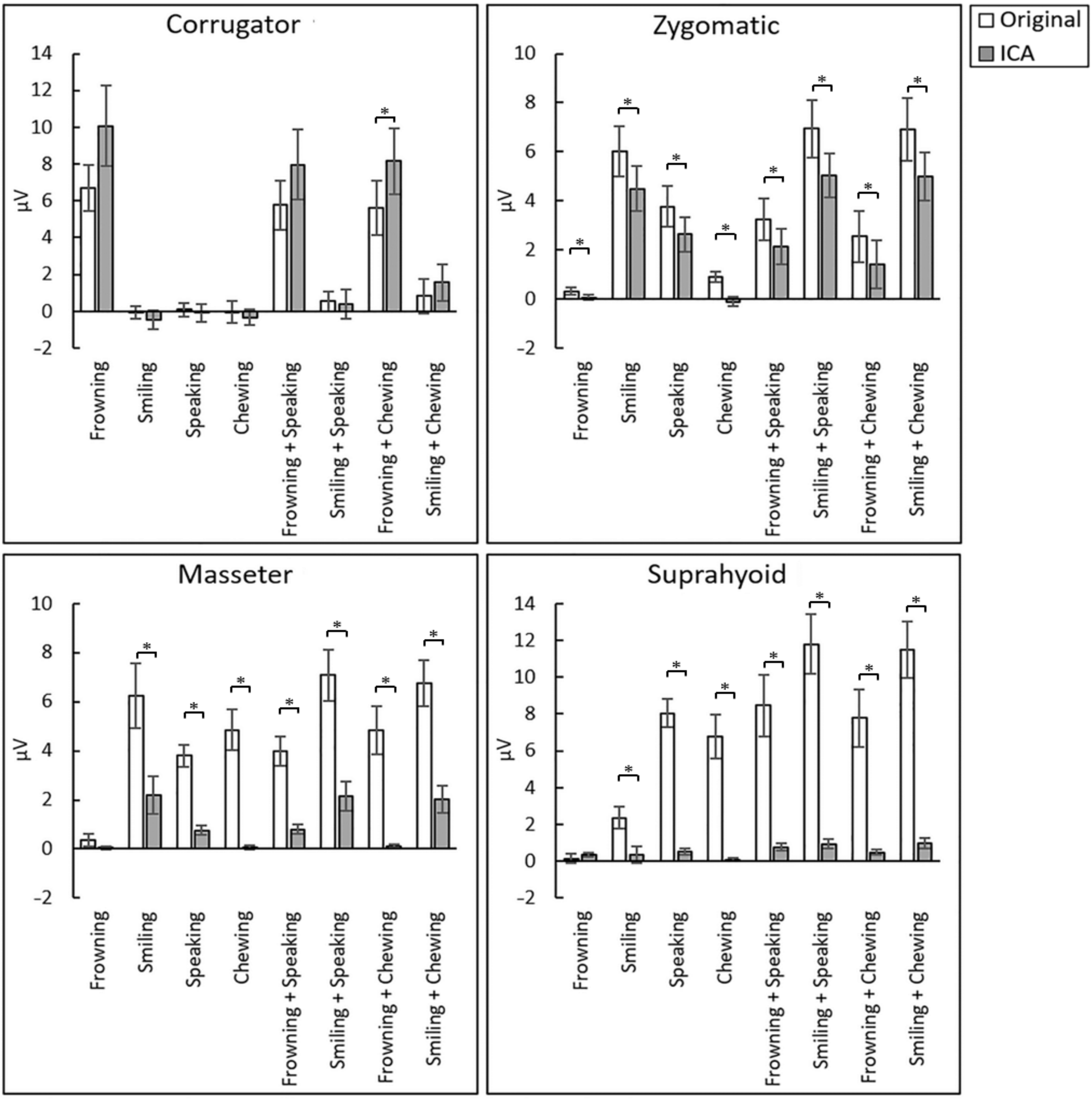

Figure S3. Mean ± standard error of original and independent component analysis (ICA)-reconstructed electromyography data indicating significant results of paired *t*-tests (two-tailed) comparing original and ICA-reconstructed signals. *, $p > 0.05$, Holm-corrected.

Table S1. Results of one-sample Wilcoxon signed-rank tests (vs. zero; two-tailed) of original electromyography signals.

Muscle	Statistic	Facial Action							
		Frowning	Smiling	Speaking	Chewing	Frowning + Speaking	Smiling + Speaking	Frowning + Chewing	Smiling + Chewing
Corrugator	*V*	**419**	257	234	307	**408**	283	**401**	**342**
	p	**< .001**	0.399	0.729	0.054	**< .001**	0.16	**< .001**	**0.007**
	r	**0.93**	0.18	0.08	0.41	**0.88**	0.30	**0.84**	**0.57**
Zygomatic	*V*	**349**	**435**	**428**	**410**	**435**	**435**	**433**	**432**
	p	**0.005**	**< .001**	**< .001**	**< .001**	**< .001**	**< .001**	**< .001**	**< .001**
	r	**0.61**	**1.00**	**0.97**	**0.89**	**1.00**	**1.00**	**0.99**	**0.99**
Masseter	*V*	**326**	**435**	**435**	**435**	**435**	**434**	**417**	**434**
	p	**0.02**	**< .001**	**< .001**	**< .001**	**< .001**	**< .001**	**< .001**	**< .001**
	r	**0.50**	**1.00**	**1.00**	**1.00**	**1.00**	**1.00**	**0.92**	**1.00**
Suprahyoid	*V*	252	**397**	**434**	**435**	**406**	**434**	**431**	**435**
	p	0.462	**< .001**	**< .001**	**< .001**	**< .001**	**< .001**	**< .001**	**< .001**
	r	0.16	**0.83**	**1.00**	**1.00**	**0.87**	**1.00**	**0.98**	**1.00**

Significant results ($p < 0.05$) corrected using Holm's method are shown in bold font.

Table S2. Results of paired Wilcoxon signed-rank tests (two-tailed) for the comparison of original and independent component analysis-reconstructed signals.

Muscle	Statistic	Facial Action							
		Frowning	Smiling	Speaking	Chewing	Frowning + Speaking	Smiling + Speaking	Frowning + Chewing	Smiling + Chewing
Corrugator	*W*	**71**	310	**346**	**356**	143	**359**	**67**	269
	p	**< .001**	0.046	**0.004**	**0.002**	0.11	**0.002**	**< .001**	0.275
	r	**0.67**	0.43	**0.59**	**0.64**	0.34	**0.65**	**0.69**	0.24
Zygomatic	*W*	**327**	**399**	**413**	**435**	**420**	**414**	**432**	**423**
	p	**0.017**	**< .001**	**< .001**	**< .001**	**< .001**	**< .001**	**< .001**	**< .001**
	r	**0.50**	**0.83**	**0.90**	**1.00**	**0.93**	**0.90**	**0.99**	**0.95**
Masseter	*W*	283	**391**	**432**	**435**	**434**	**429**	**417**	**415**
	p	0.162	**< .001**	**< .001**	**< .001**	**< .001**	**< .001**	**< .001**	**< .001**
	r	0.30	**0.80**	**0.99**	**1.00**	**1.00**	**0.97**	**0.92**	**0.91**
Suprahyoid	*W*	133	**349**	**435**	**435**	**406**	**435**	**432**	**435**
	p	0.069	**0.004**	**< .001**	**< .001**	**< .001**	**< .001**	**< .001**	**< .001**
	r	0.39	**0.61**	**1.00**	**1.00**	**0.87**	**1.00**	**0.99**	**1.00**

Significant results ($p < 0.05$) corrected using Holm's method are shown in bold font.
